# Skp1 in lung cancer: clinical significance and therapeutic efficacy of its small molecule inhibitors

**DOI:** 10.18632/oncotarget.5547

**Published:** 2015-10-12

**Authors:** Yong-Qiang Liu, Xiao-Lu Wang, Xin Cheng, Yong-Zhi Lu, Gui-Zhen Wang, Xin-Chun Li, Jian Zhang, Zhe-Sheng Wen, Zhi-Liang Huang, Qin-Lei Gao, Li-Na Yang, Yong-Xian Cheng, Sheng-Ce Tao, Jinsong Liu, Guang-Biao Zhou

**Affiliations:** ^1^ State Key Laboratory of Membrane Biology, Institute of Zoology, Chinese Academy of Sciences & University of Chinese Academy of Sciences, Beijing 100101, China; ^2^ State Key Laboratory of Respiratory Disease, Guangzhou Institute of Biomedicine and Health, Chinese Academy of Sciences, Guangzhou 510530, China; ^3^ School of Life Sciences, Anhui University, Hefei 230039, China; ^4^ Department of Thoracic Surgery, The Cancer Hospital, Sun Yat-Sen University, Guangzhou 510080, China; ^5^ State Key Laboratory of Phytochemistry and Plant Resources in West China, Kunming Institute of Botany, Chinese Academy of Sciences, Kunming 650201, China; ^6^ Shanghai Center for Systems Biomedicine, Key Laboratory of Systems Biomedicine (Ministry of Education), Shanghai Jiao Tong University, Shanghai 200240, China

**Keywords:** Skp1, lung cancer, structure-based high-throughput virtual screening, inhibitors, 6-O-angeloylplenolin

## Abstract

Skp1 is an essential adaptor protein of the Skp1-Cul1-F-box protein complex and is able to stabilize the conformation of some ubiquitin E3 ligases. However, the role Skp1 plays during tumorigenesis remains unclear and Skp1-targeting agent is lacking. Here we showed that Skp1 was overexpressed in 36/64 (56.3%) of non-small cell lung cancers, and elevated Skp1 was associated with poor prognosis. By structure-based high-throughput virtual screening, we found some Skp1-targeting molecules including a natural compound 6-*O*-angeloylplenolin (6-OAP). 6-OAP bound Skp1 at sites critical to Skp1-Skp2 interaction, leading to dissociation and proteolysis of oncogenic E3 ligases NIPA, Skp2, and β-TRCP, and accumulation of their substrates Cyclin B1, P27 and E-Cadherin. 6-OAP induced prometaphase arrest and exerted potent anti-lung cancer activity in two murine models and showed low adverse effect. These results indicate that Skp1 is critical to lung cancer pathogenesis, and Skp1 inhibitor inactivates crucial oncogenic E3 ligases and exhibits significant therapeutic potentials.

## INTRODUCTION

The S phase kinase-associated protein 1 (Skp1)–Cullin 1 (Cul1)–F-box protein (SCF) complexes are multi-protein E3 ubiquitin ligase complexes that promote the ubiquitination and degradation of a large number of regulatory proteins involved in diverse processes [1]. Accumulative evidence demonstrates that components of SCF complexes play pivotal roles in tumorigenesis [2]. For example, Cul1 is increased in melanoma and breast and lung cancers, and is able to promote cancer cell proliferation [3, 4, 5]. Many of the F box proteins function as oncoproteins (e.g., Skp2, NIPA and β-TRCP) or tumor suppressors (e.g., Fbxw7) [1, 2]. Skp2 is overexpressed in human cancers [6] and is able to promote degradation of p27 [7] and activation of Akt, leading to cancer initiation and progression [8, 9]. β-TRCP activates NFκB by mediating ubiquitination and degradation of IκBα [10], and enhances β-Catenin transcriptional activity [11]. Skp1 is the essential adaptor protein linking the F-box protein and Cul1 [12, 13, 14, 15]. However, the expression of Skp1 and its roles in carcinogenesis remain largely unknown.

Some F-box proteins have been shown to be rational targets for the treatment of human cancers. For example, Skp2 targeting suppresses tumorigenesis [16] and induces apoptosis of cancer cells [17]. Compounds which inhibit Skp2-mediated p27 degradation promote cell-type specific blockage in the G1 or G2/M phases [18, 19, 20]. Compound #25 [21] binds Skp2 and prevents Skp2-Skp1 interaction, leading to the inhibition of Skp2 activity and suppression of cancer stem cells. Inhibition of β-TRCP suppresses breast cancer cells [22], while elevation of Fbxw7 reverses resistance to chemotherapies [23]. However, whether Skp1 could serve as a therapeutic target is yet to be determined. We hypothesized that Skp1 could be a potential target, because it is a prerequisite for the functions of some oncogenic E3 ligases including Skp2 [24], Fbxo44 [25], Fbxo6, Fbxo1717, Fbxo27 [26] and the nuclear interaction partner of ALK (NIPA) that targets cyclin B1 for ubiquitination during interphase and prevents premature mitotic entry [27, 28, 29].

In this study, we assessed the expression of Skp1 in non-small cell lung cancers (NSCLCs), and screened for Skp1 inhibitors in a total of 21,008 compounds by structure-based high-throughput virtual screening. We showed that Skp1 was overexpressed in 36/64 (56.3%) of NSCLCs and was inversely associated with patients’ prognosis. We found some compounds that could bind Skp1 at two pockets. Among these molecules, a natural compound 6-*O*-angeloylplenolin (6-OAP) that was isolated from a medicinal herb *Centipeda minima*, could bind and sequester Skp1, resulting in dissociation and degradation of Skp2, NIPA, and β-TRCP, but did not perturb the expression of Fbxw7. 6-OAP showed potent *in vitro* and *in vivo* anti-lung cancer activity with minimal adverse effect, demonstrating the therapeutic potentials of Skp1 inhibitors.

## RESULTS

### Skp1 is overexpressed and inversely associated with prognosis in lung cancer

**Figure 1:**
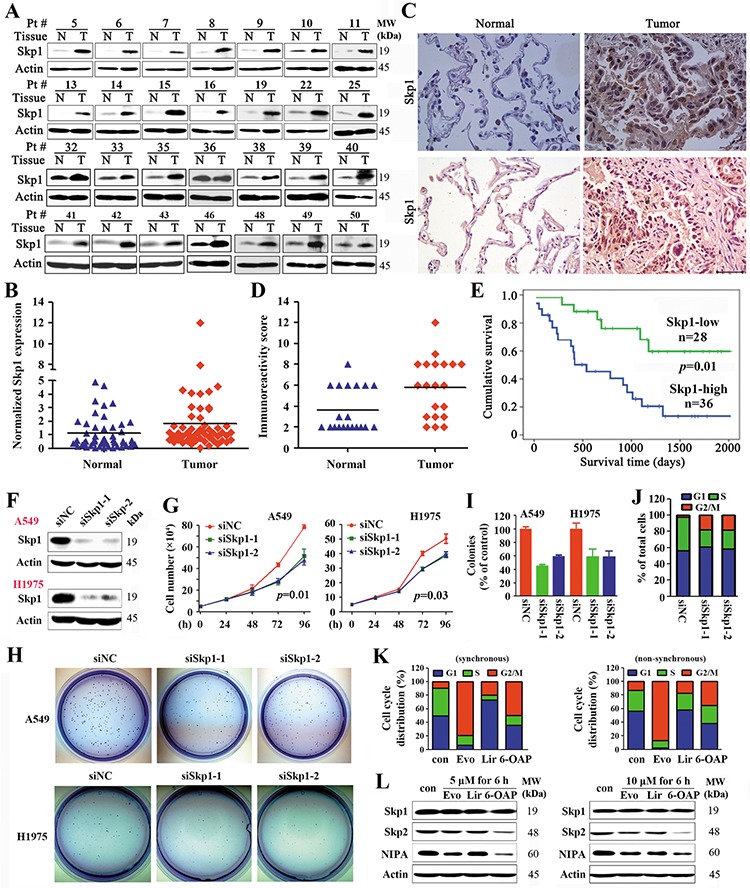
Skp1 in lung cancer **A.** Representative Western blot analyses of lysates of tumor and adjacent normal lung tissues harvested from NSCLCs (*n* = 64). **B.** The densitometry analysis of the Western blot results. **C.** Immunohistochemistry of Skp1 in NSCLCs using an anti-Skp1 antibody. Size bar, 50 μm. **D.** The immunoreactivity score was calculated. **E.** Overall survival of the 64 patients. **F–J.** A549 and H1975 cells were transfected with Skp1 specific siRNAs (F), the cell proliferation were analyzed by trypan blue exclusion analyses (G), and the clonogenic activity of cells was tested by the Flat plate clone formation assay (H, I). The cell cycle distribution of H1975 cells were analyzed (J). **K, L.** Effects of three Skp1-targeting compounds on lung cancer cells. The compounds were identified by structure-based high-throughput virtual screening for Skp1 inhibitors (See also Figure S1). Synchronous or asynchronous H1975 cells were treated with or without the compounds, and cell cycle distribution was determined (K). Western blot analysis of lysates of the cells treated with indicated compounds (L). Evo, Evodiamine; Lir, Liriodenine; 6-OAP, 6-*O*-angeloylplenolin.

We tested the expression of Skp1 in 64 previously untreated NSCLCs (Table 1) by Western blot (Figure 1A, 1B) and immunohistochemistry (Figure 1C, 1D), and showed that in 36 (56.3%) of the patients the expression of Skp1 was significantly higher in tumor samples than their adjacent normal lung tissues. The densitometry analyses of the Western blot bands and the immunoreactivity score of immunohistochemistry confirmed the elevation of Skp1 in tumor samples (Figure 1B, 1D). Importantly, patients with higher levels of Skp1 had much shorter overall survival than those with lower Skp1 expression (*p* = 0.01; Figure 1E).

**Table 1:** Summary of baseline demographic characteristics of the 64 patients

Characteristics	Cases	Skp1-high, *n* (%)	*P* values
Total	64	36 (56.3)	
Gender			0.68
Male	44	24 (54.6)	
Female	20	12 (60)	
Age			
<65	46	26 (56.5)	0.94
≥65	18	10 (55.6)	
Smoking			0.7
Smoker	36	21 (58.3)	
Non-smoker	28	15 (53.6)	
Histology			0.76
Adenocarcinoma	39	22 (56.4)	
Squamous cell carcinoma	21	11 (52.4)	
Adenosquamouscarcinoma	3	2 (66.7)	
Small cell lung cancer	1	1	
TNM stage			0.15
I	29	14 (48.3)	
II	8	4 (50)	
III	21	15 (71.4)	
IV	6	3 (50)	

### Silencing Skp1 exerts inhibitory effects on lung cancer cells

The potential role of Skp1 in lung cancer was evaluated by siRNA-mediated silencing in NSCLC lines A549 and NCI-H1975 (harboring the L858R/T790M-EGFR) [30], and the results showed that knockdown of Skp1 (Figure 1F) led to a significant inhibition of cell growth/proliferation (Figure 1G) and suppression of colony forming activity (Figure 1H, 1I) of the cells. We tested the effect of Skp1 knockdown on cell cycle distribution, and found that in asynchronous H1975 cells, siRNA-mediated Skp1 silencing resulted in G2/M phase arrest (Figure 1J). These results indicate that Skp1 is critical to lung cancer cell proliferation.

### Structure-based high-throughput virtual screening for Skp1 inhibitors

We screened for specific Skp1 small molecule inhibitors by structure-based high-throughput virtual screening [31] using the Skp1 model from the crystal structure of Skp1-Skp2-Cks1 in complex with a p27 peptide (PDB accession 2AST) [32] as the receptor in the molecular docking. Two libraries were used for the screening: library 1 contained 1008 natural compounds and library 2 was a commercial library composed of 20,000 compounds. The poses docked to Skp1 were clustered into two potential binding pockets (Figure S1A), pocket 1 (P1; comprised of residues Q97, L100, F101, I104, V123, F139 and I141; Figure S1B) and pocket 2 (P2; comprised of residues I135, R136, I141, N143, D144, E150, V153 and N157; Figure S1C). P1 accommodates the residue W97 while P2 accommodates the residues from L114 to K125 of Skp2. Residue N143 of Skp1 forms an H-bond with V123 of Skp2 (mediated by a water molecule), and N157 of Skp1 forms two H-bonds with Ser121 and Leu118 of Skp2. When docked to site P1, the interactions between ligands and Skp1 mainly included H-bond with Q97, Pi stacking interaction with F101 and F139, and hydrophobic interaction. When docked to site P2, most compounds can form H-bond with residues R136 and N143 in Skp1. Some compounds can form additional H-bonds with other residues, such as D144, F145, E150 and N157. Residue N143 of Skp1 forms an H-bond with V123 of Skp2 (mediated by a water molecule), and N157 of Skp1 forms two H-bonds with Ser121 and Leu118 of Skp2.

The binding affinity energy (BAE) of the compounds to Skp1 was calculated, and compounds with BAE ≤ − 7.0 kcal/mol were further filtered by the Lipinski's rule of five drug-like property [33] and ADME/Tox prediction, resulting in identification of 28 lead compounds (Figure S1D, Table S1). We tested the effects of these compounds on cancerous and normal lung epithelial cell lines, and found that three natural compounds, liriodenine, evodiamine, and 6-OAP had significant BAE to Skp1 and inhibitory effects on lung (A549, H1975 and H460), gastric (BGC823), breast (MCF7), and liver (BEL7402) cancer cell lines (Table S1). We tested the effects of these 3 compounds on cell cycle distribution, and found that evodiamine and 6-OAP inhibited G2/M while liriodenine inhibited G1 phase of synchronous and non-synchronous A549 cells (Figure 1K). At protein level, these compounds did not down-regulated the expression of Skp1. Interestingly, 6-OAP down-regulate the expression of Skp2 and NIPA, while evodiamine slightly suppressed the expression of these two proteins (Figure 1L). We therefore investigated the mechanisms of action of 6-OAP on Skp1 and cell cycle inhibition.

### 6-OAP is a Skp1 inhibitor

**Figure 2:**
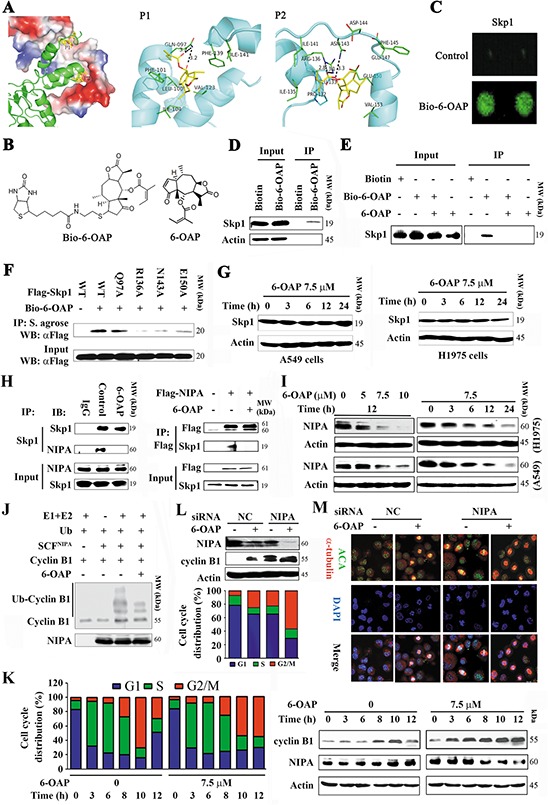
6-OAP directly binds Skp1 and interferes with SCF^NIPA^ **A.** Two potential binding pockets (P1 and P2) of Skp1 for 6-OAP, revealed by docking 6-OAP to Skp1 (PDB code: 2AST). In the left panel, Skp2 (shown as cartoon) interacts with Skp1 (shown as surface) via P1 and P2, and only Skp1 is used during the docking. In the middle and right panels, 6-OAP (shown as sticks) is predicted to interact with Skp1 (shown as cartoon) via P1 and P2, respectively. **B.** Chemical structure of 6-OAP and Bio-6-OAP. **C.** Images of Bio-6-OAP-Skp1 interaction on the slides. **D.** H1975 cells were treated with Biotin or Bio-6-OAP at 50 μM for 6 h, lysed, and the cell lysates were subjected to immunoprecipitation using streptavidin agarose and Western blot using indicated antibodies. **E.** H1975 cells were treated with Bio-6-OAP (50 μM) in the presence or absence of 6-OAP (100 μM) for 6 h, lysed, and the cell lysates were subjected to immunoprecipitation and Western blot. **F.** 293T cells were transfected with wild type (WT) or mutant *Skp1* for 48 h, lysed, the lysates were subjected to immunoprecipitation using streptavidin (S.) agarose and Western blot using indicated antibodies. **G.** The cells were treated with 6-OAP, lysed, and subjected to Western blot. **H.** H1975 cells were treated with or without 6-OAP for 3 h, lysed, and immunoprecipitation and Western blot assays were performed (left panel). 293T cells were transfected with pcDNA3.1-*flag-NIPA*, treated with or without 6-OAP, and lysed for immunoprecipitation and Western blot (right panel). **I.** Cells were treated with 6-OAP, lysed, and Western blot was performed. **J.** An *in vitro* ubiquitination assay using SCF^NIPA^, Cyclin B1, and 6-OAP. **K.** A549 cells were synchronized to G1/S boundary and released, and treated with or without 6-OAP. Cell cycle distribution was determined (left), and the expression of NIPA and Cyclin B1 was analyzed by Western blot (right). **L.** A549 cells transfected with control or *NIPA* specific siRNA were treated with 6-OAP for 12 h, harvested for Western blot (upper) or flow cytometry analysis (lower). **M.** A549 cells transfected with *NIPA*-specific siRNA were treated with or without 6-OAP (7.5 μM) for 12 h, and analyzed by immunofluorescence labeling with anti-centromere sera, anti-α-tubulin antibody, and DAPI. Size bar, 5 μm.

The computational docking results showed that 6-OAP could form two H-bonds with R136 and one H-bond with N143, and closely contacted with E150 and interacted with the hydrophobic residues at P2 of Skp1. 6-OAP could be docked onto the P1 pocket, forming two H-bonds with Q97 and Pi-Pi interaction with F101, and its hydrophobic group could form hydrophobic interaction with residues in P1 pocket (Figure 2A). By interacting with either the P1 or P2 pocket, 6-OAP could interfere with the interaction between Skp2 and Skp1.

To confirm 6-OAP/Skp1 interaction, biotinylated 6-OAP (Bio-6-OAP, Figure 2B) was synthesized and validated by mass spectrum (Figure S2) and nuclear magnetic resonance, with a purity of 95% determined by HPLC. The purified Skp1 protein was printed on slides and incubated with Bio-6-OAP followed by treatment with a Cy3 conjugated streptavidin (Cy3-SA), and the Bio-6-OAP-Skp1 interaction was detected (Figure 2C). In Bio-6-OAP- treated H1975 cells, Skp1 was pulled down by streptavidin agarose (Figure 2D). The *in vitro* experiment showed that the binding of Bio-6-OAP to Skp1 could be markedly attenuated by unlabeled 6-OAP (Figure 2E), confirming the direct binding of 6-OAP to Skp1.

Docking analysis suggested that residues Q97, N143, R136 and E150 of Skp1 were involved in the binding with 6-OAP (Figure 2A). To confirm whether these residues were critical for the 6-OAP interaction, site-directed mutagenesis on Skp1 was performed, and plasmids containing wild type (WT) or mutant *Skp1* were transfected into A549 cells to purify Skp1 protein for *in intro* binding analysis. We showed that while WT Skp1 strongly recruited 6-OAP, Q97A mutation only slightly attenuated the binding affinity; however, R136A, N143A, and E150A mutations drastically inhibited Skp1 from binding to 6-OAP (Figure 2F), indicating that the P2 pocket of Skp1 is critical for 6-OAP binding. Of note, treatment of A549 and H1975 cells with 6-OAP did not perturb the expression of Skp1 at protein level (Figure 2G), suggesting that 6-OAP does not affect Skp1 expression, but might sequestrate it and therefore interfere with Skp1-F-box protein binding affinity.

### 6-OAP targets the SCF^NIPA^ complex

Skp1 can bind and thus stabilize NIPA [28] which ubiquitinates Cyclin B1 and regulates mitotic entry [27]. We examined whether or not 6-OAP could dissociate Skp1-NIPA interaction by immunoprecipitation and Western blot assays, and found that in H1975 cells upon 6-OAP treatment, Skp1-NIPA binding affinity was markedly reduced (Figure 2H, left panel); in 293T cells transfected with *Flag-NIPA*, 6-OAP also markedly suppressed Skp1-NIPA interaction (Figure 2H, right panel). We showed that in A549 and H1975 cells, 6-OAP caused down-regulation of NIPA in a dose- and time-dependent manner (Figure 2I). In an *in vitro* ubiquitination assay, we showed that SCF^NIPA^ was able to ubiquitinate Cyclin B1, while 6-OAP treatment inhibited this effect (Figure 2J). We then examined the kinetics of mitotic entry and NIPA expression in A549 cells exposed to 6-OAP. To do this, A549 cells were synchronized at G1/S boundary site by thymidine treatment. We showed that the cells entered into mitosis in 8 h, and the 6-OAP-treated cells arrested in M phase while untreated cells exited in 12 h (Figure 2K, left panel). Accordingly, NIPA was down-regulated while Cyclin B1 was up-regulated in cells treated with 6-OAP for 12 h (Figure 2K, right panel). When the expression of NIPA was reduced to approximately 50% by 6-OAP treatment or NIPA-specific siRNA, approximately equal proportion (25% versus 22%) of the cells were arrested at M phase; when NIPA was down-regulated by about 90% by combinatory treatment, 60% of the cells were arrested at M phase (Figure 2L). Immunofluorescence analysis showed that both 6-OAP treatment and NIPA knockdown induced mitotic arrest (Figure 2M). These results suggested that 6-OAP dissociated NIPA from Skp1 and triggered its proteolysis, leading to accumulation of Cyclin B1 in lung cancer cells.

### Skp1–6-OAP binding dissociates F-box proteins Skp2, β-TRCP and Fbxw7

**Figure 3:**
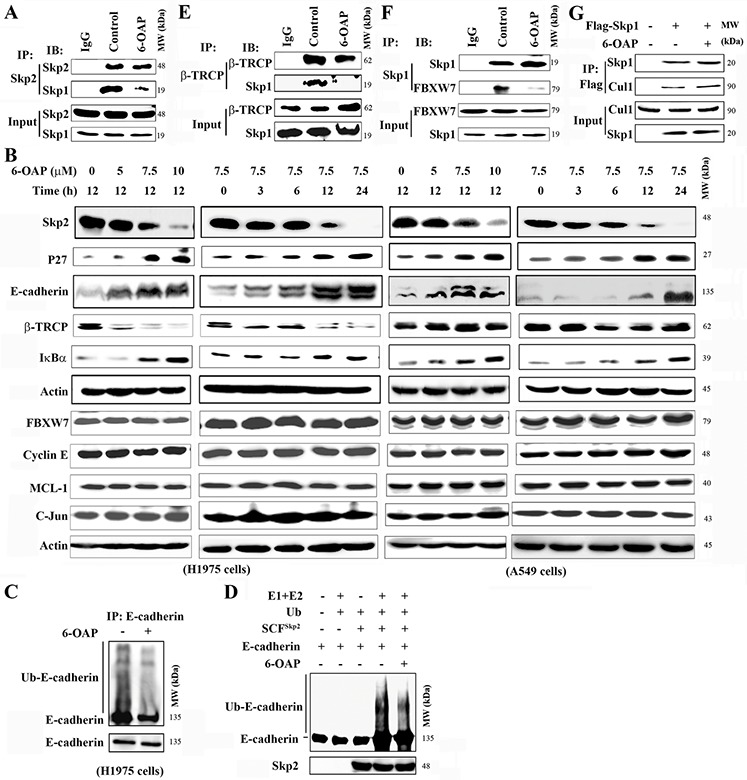
Effects of 6-OAP on Skp2, β-TRCP, Fbxw7 and their substrate proteins **A.** H1975 cells were treated with or without 6-OAP for 3 h, lysed, and immunoprecipitation and Western blot were performed using indicated antibodies. **B.** The cells were treated with 6-OAP, lysed and subjected to Western blot. **C, D.** 6-OAP inhibited ubiquitination of E-Cadherin, revealed by immunoprecipitation and Western blot in H1975 cells upon 6-OAP (C) and an *in vitro* ubiquitination assay using SCF^Skp2^, purified E-Cadherin and 6-OAP (D). **E, F.** H1975 cells were treated with 6-OAP, lysed, and analyzed by immunoprecipitation and Western blot. **G.** 293T cells were transfected with Flag-Skp1 and treated with 6-OAP, lysed, and the lysates were subjected to immunoprecipitation and Western blot assays.

The 6-OAP binding sites in Skp1 located in the core part of Skp1-Skp2 interface [24]. We tested whether or not 6-OAP dissociated Skp2 from Skp1 by co-immunoprecipitation, and found that in A549 cells, Skp1-Skp2 binding affinity was high, while 6-OAP dramatically attenuated this interaction and released Skp2 (Figure 3A). We assessed the expression of Skp2 at protein level, and found that in H1975 and A549 cells treated with 6-OAP, Skp2 was down-regulated in a dose- and time-dependent manner (Figure 3B). On the contrary, two substrate proteins of Skp2 which are involved in regulation of G2-M progression, P27 [18] and E-cadherin [34], were up-regulated in a dose- and time-dependent fashion (Figure 3B). In H1975 cells, 6-OAP treatment suppressed ubiquitination of E-cadherin (Figure 3C); in an *in vitro* ubiquitination assay, 6-OAP inhibited Skp2-induced ubiquitination of E-cadherin (Figure 3D). These results indicate that Skp2 function is inhibited by 6-OAP.

We tested the effects of 6-OAP on other Skp1 binding partners, and found that this compound perturbed the Skp1-F-box protein interaction and dissociated β-TRCP (Figure 3E) and Fbxw7 (Figure 3F), but did not affect Skp1-Cul1 binding affinity (Figure 3G). In H1975 and A549 cells, 6-OAP induced downregulation of β-TRCP and up-regulation of its substrate protein IκBα, and did not interfere with the expression of Fbxw7 and its substrates Cyclin E, MCL-1 and C-Jun (Figure 3B).

### Skp1 expression is associated with the 6-OAP sensitivity of the cells

**Figure 4:**
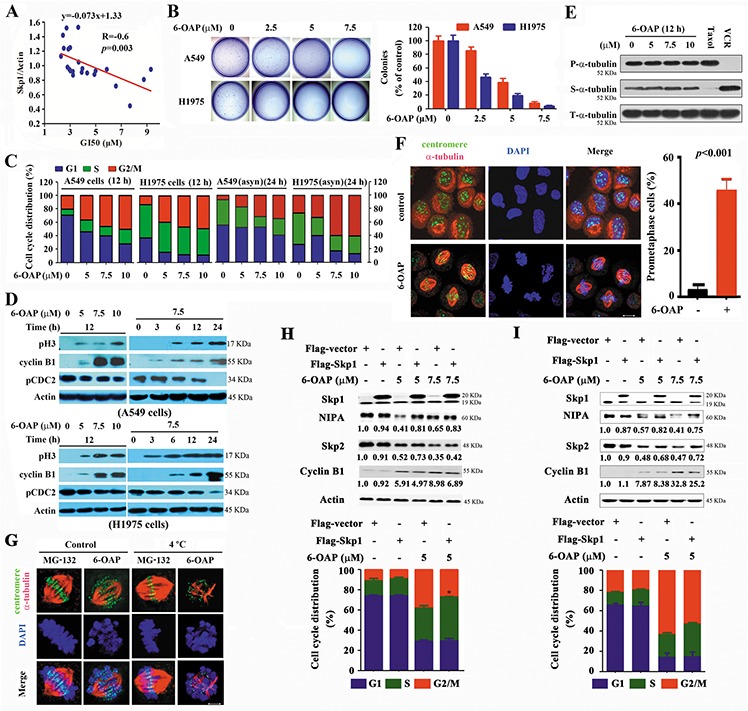
6-OAP induces prometaphase arrest in lung cancer cells **A.** The GI50s of 6-OAP in cells is associated with the relative Skp1 expression. **B.** Effects of 6-OAP on clonogeinc activity of lung cancer cells. **C.** The synchronized or asynchronous (asyn) to G1/S boundary cells were treated with 6-OAP at indicated concentrations for 12 h. Cell cycle distribution was determined by propidium iodide (PI) staining and flow cytometry analysis. **D.** The cells were treated with 6-OAP, lyzed, and Western blot was performed using antibodies indicated. **E.** The cells were treated with 6-OAP, taxol (50 nM) or Vincristine (VCR; 50 nM) for 12 h. The polymerized (P) and soluble (S) tubulin fractions were prepared and subjected to Western blot using anti-α-tubulin antibody. T-α-tubulin, total-α-tubulin. **F.** A549 cells were treated with 7.5 μM 6-OAP for 12 h, and assayed by immunofluorescence labeling with anti-centromere sera (green), anti-α-tubulin antibody to visualize microtubules (red), and DAPI to counter stained DNA (blue). Size bar, 5 μm. **G.** A549 cells were treated with 7.5 μM 6-OAP for 12 h or 10 μM MG-132 for 3 h, and incubated in ice-cold media for 10 min and stained with anti-centromere sera, anti-α-tubulin antibody, and DAPI. **H, I.** The A549 (H) and 293T (I) cells were transfected with *Skp1*, synchronized at G1/S boundary site by thymidine treatment, and treated with 6-OAP for 12 hours. The cells were analyzed by flow cytometry to evaluate the cell cycle distribution, or lysed for Western blot analysis. Numbers under the NIPA, Skp2, and Cyclin B1 bands are the relative expression values to Actin determined by densitometry analysis. *p = 0.04.

We tested the effects of 6-OAP on 21 cancer cell lines (including lines of lung, liver, gastric, breast, kidney cancers, leukemia, and myeloma) and 2 normal cell lines (16HBE and HLF) by MTT assay, and found that 6-OAP inhibited the proliferation of these cell lines with the GI50s ranged from 2.29 to 9.31 μM (Table S2, Figure S3A), and the Bio-6-OAP retained the proliferation inhibition activity (Figure S3B). To assess the potential association between the Skp1 expression and the sensitivity of the cells to 6-OAP, the Western blot was performed (Figure S3C) and the densitometry analysis of Western blot bands was conducted to evaluate the relative Skp1 expression: Relative Skp1 expression=Skp1/β-Actin (Table S2). The logistic regression analysis was performed, and the results showed that Skp1 expression was associated with 6-OAP sensitivity of the cells (R = −0.6, *p* = 0.003, Figure 4A). 6-OAP also significantly inhibited the growth (Figure S3D) and clonogenic activity of A549 and H1975 cells (Figure 4B).

### 6-OAP arrests mitosis at prometaphase in lung cancer cells

Cell cycle distribution was analyzed in double-thymidine synchronized cells, and the results showed that treatment with 6-OAP at 5 to 10 μM for 12 h accumulated A549 and H1975 cells in G2/M phase (Figure 4C). 6-OAP also arrested cell cycle at G2/M phase in asynchronous cells (Figure 4C). G2/M phase blockade was also seen in Bio-6-OAP treated cells (Figure S3E–S3G). However, apoptotic effect of 6-OAP on lung cancer cells was not dramatic (Figure S3H).

The mitotic-specific phosphorylation of histone 3 at Ser10 (pH3) and the activation of cyclin B1/Cdc2 complex were analyzed. We showed that 6-OAP up-regulated pH3 and cyclin B1 and down-regulated the tyrosine-15-phosphorylated Cdc2 (pCdc2 (Y15)) in A549 and H1975 cells (Figure 4D), indicating that 6-OAP arrests lung cancer cells in an early stage of mitosis. However, 6-OAP did not interfere with polymerization of tubulins (Figure 4E). We performed immunofluorescence staining of microtubule and chromosome, and found that 6-OAP treatment for 12 h significantly increased prometaphase cells characterized by bipolar spindle assembly and chromosome compression, with a number of chromosomes remained at the poles and did not align at the spindle equator (Figure 4F). We analyzed the stability of kinetochore microtubules by cold-induced depolymerization, and observed that the cells treated with proteasome inhibitor MG-132 [35] at 4°C arrested at metaphase and formed stable kinetochore microtubules (Figure 4G). However, a subset of kinetochores unattached to the spindle poles were observed in 6-OAP-treated cells (Figure 4G). These results demonstrate that cells treated with 6-OAP are arrested at prometaphase.

### Skp1 is critical to 6-OAP-induced-mitotic arrests

To test the role of Skp1 in cell cycle arrest, the cells were transfected with Skp1, synchronized at G1/S boundary site by thymidine treatment, and treated with 6-OAP for 12 hours. We found that in A549 cells, 6-OAP downregulated NIPA and Skp2 and upregulated Cyclin B1, and arrested cell cycle at G2/M phase. Transfection of *Skp1* antagonized these effects (Figure 4H). These results were also observed in 293T cells (Figure 4I), demonstrating the roles of Skp1 in 6-OAP-induced mitotic arrest.

### *In vivo* anti-lung cancer activity of 6-OAP

**Figure 5:**
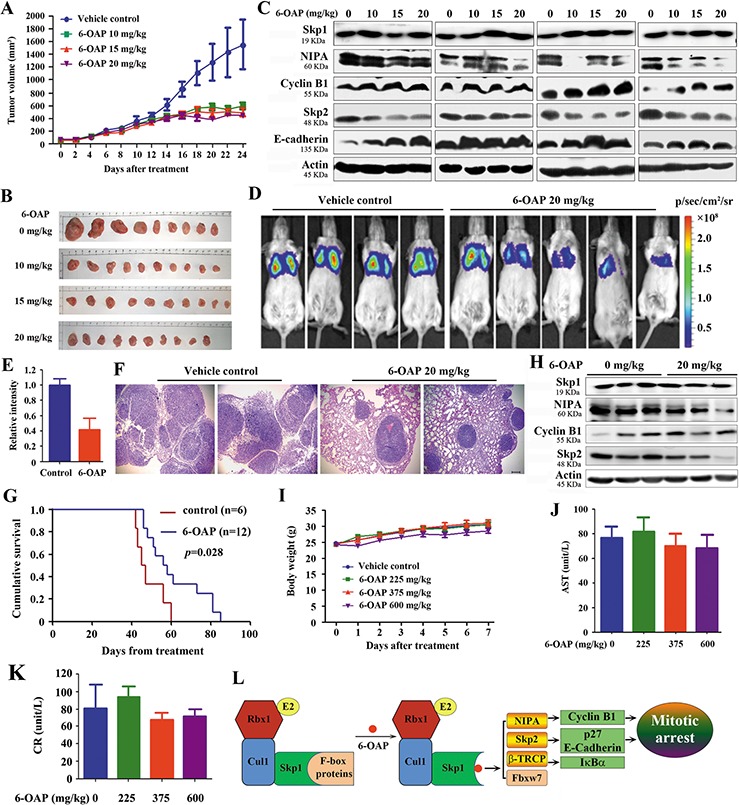
*In vivo* anti-lung cancer efficacy and acute toxicity of 6-OAP **A.** Nude mice subcutaneously inoculated with H1975 cells were treated with 6-OAP or vehicle for 24 days, and tumor volume was estimated every two days. **B.** Images of xenograft tumors obtained from the mice. **C.** Western blot assays using lysates of isolated tumors and indicated antibodies. **D.** A549-Luciferase cells were intravenously injected into SCID mice, and 1 week later the mice were randomized to receive vehicle (*n* = 6) or 6-OAP treatment (*n* = 12). The mice were detected by IVIS Spectrum. **E.** The relative luciferase intensity in the mice. **F.** Lung tissue sections of mice treated with 6-OAP or vehicle control were stained with hematoxylin-eosin and analyzed using a research microscope. Size bar, 100 μm. **G.** Life span of the mice. **H.** Western blot analysis using lysates of tumor samples isolated from the mice. **I.** Intravenous injection of 6-OAP at 225 to 600 mg/kg did not significantly affect the body weight of Kunming mice. **J, K.** The serum AST (J) and CR (K) levels were detected after intravenous injection of 6-OAP or vehicle in Kunming mice. **L.** A schematic representation of mechanisms of 6-OAP-induced mitosis arrest.

In nude mice (*n* = 10 for each group) subcutaneously inoculated with H1975 cells (2 × 10^6^), intraperitoneal injection of 6-OAP at 10 to 20 mg/kg (once per day for 24 days) significantly inhibited tumor growth (Figure 5A and 5B), but did not reduce body weight of the mice (Figure S3I). 6-OAP treatment did not perturb Skp1 expression, but down-regulated NIPA and Skp2, and up-regulated Cyclin B1 and E-cadherin in tumor samples (Figure 5C). In SCID/Beige mice n=6 for control group and n=12 for 6-OAP treatment group injected with A549-luciferase cells (1 × 10^6^), 20 mg/kg 6-OAP (intravenously injection, once every two days for 30 days) significantly suppressed tumor growth reflected by decrease of luciferase bioluminescence intensity (Figure 5D, 5E). 6-OAP reduced dissemination of disease and prevented destruction of tissue architectures (Figure 5F), and prolonged life span of the mice (Figure 5G). 6-OAP also induced downregulation of NIPA and Skp2 and up-regulation of Cyclin B1 in tumor samples (Figure 5H).

We tested the adverse effects of 6-OAP, and showed that normal Kunming mice receiving intravenously injection of 6-OAP at 225–600 mg/kg survived during one week's observation, and did not show body weight loss (Figure 5I). These mice had normal serum concentrations of aminotransferase (AST) and creatinine (CR) (Figure 5J, 5K).

## DISCUSSION

Recent studies have uncovered the important roles of SCF complex components in carcinogenesis. For example, overexpressed Cul1 promotes cancer cell proliferation and predicts poor prognosis, while alterations in the Cul1 neddylation/deneddylation pathway contributes to the development of highly aggressive lung tumors [36, 3, 4, 5]. Oncogenic E3 ligases promote degradation of tumor suppressors and contribute to uncontrolled proliferation, genomic instability, and malignant transformation [2]. Here we showed that Skp1 was overexpressed in lung cancers and was inversely associated with clinical outcome (Figure 1), while knockdown of Skp1 resulted in inhibition of cancer cell proliferation and clonogenic activity (Figure 1). Skp1 is required for stabilization of some F-box proteins [26, 28]. We showed that dissociation of NIPA, Skp2 and β-TRCP from Skp1 led to their degradation (Figure 2–4). Therefore, the elevated Skp1 might facilitate oncogenic E3 ligases and their downstream molecules such as NFκB [10] and β-Catenin [11], promoting malignant transformation of lung epithelial cells.

We aimed to identify Skp1 inhibitors and evaluate their therapeutic potentials, and found some compounds that could bind Skp1 at P1 and P2 pockets by H-bonds, Pi stacking interactions, and hydrophobic interactions (Figure S1). The representative three molecules (evodiamine, 6-OAP, and liriodenine) exhibited moderate to potent cytotoxicity against several cancer cell lines. These compounds perturbed the expression of oncogenic E3 ligases NIPA and Skp2, but did not affect Skp1 expression (Figure 1). 6-OAP is extracted from a medicinal herb *Centipeda minima* (L.) which is rich in many countries including China and Korea [37]. We showed that 6-OAP could bind Skp1 mainly at P2 pocket, with R136, N143 and E150 as the direct binding amino acids. This region locates in the core part of Skp1-Skp2 interface [24], and Skp1–6-OAP interaction competitively dissociated E3 ligases Skp2, NIPA, and β-TRCP from Skp1 (Figures 2 and 3). Skp1 is able to stabilize the conformation of NIPA [28] and other F-box proteins [26], whereas sequestration of Skp1 by 6-OAP induced proteolysis of NIPA, Skp2 and β-TRCP, and up-regulation of their substrate proteins Cyclin B1, P27, E-Cadherin, and IκBα (Figure 2, 3 and 5L). Interestingly, 6-OAP did not interfere with Skp1-Cul1 interaction, while Fbxw7 was dissociated but not subsequently degraded and its oncogenic substrates Cyclin E, MCL-1 and C-JUN were not up-regulated (Figure 3). The mechanism underlying these observations remains an open question.

Mitosis is a complex process in actively proliferating cells, resulting in the division of a cell into two genetically identical daughter cells. In eukaryocytes, the anaphase-promoting complex (APC) attaches ubiquitin to cyclin B1 [38, 39], and the proteasomal degradation of cyclin B1 can be inhibited by the spindle-assembly checkpoint (SAC) which is comprised of checkpoint proteins including Bub1, BubR1/Mad3, Bub3, Mad1 and Mad2 [40]. Huang et al [41] showed that blocking mitotic exit by silencing Cdc20, a co-factor of APC, slowed Cyclin B1 proteolysis and thus allowed more time for death initiation. Sackton et al [42] recently showed that simultaneously disrupting the protein–protein interactions within the APC/C–Cdc20–substrate ternary complex could synergistically inhibit APC/C-dependent proteolysis and mitotic exit. We previously reported that 6-OAP induced BubR1 assemble and enhanced the binding affinity between Mad2 and Cdc20, but the role APC played in 6-OAP-induced mitotic arrest remained unclear since 6-OAP was unable to directly target APC or SAC component [43]. In this study, we showed that 6-OAP inactivated NIPA and accumulated Cyclin B1 in lung cancer cells (Figure 2). In HeLa cells, the mitotic block upon NIPA knockdown was presumably not cyclin B related, but rather a result of the accumulation of other yet-to-be-identified NIPA targets [27]. In myeloma cells, however, cyclin B1 silencing abrogated 6-OAP-induced mitotic arrest [43]. Moreover, 6-OAP treatment dissociated Skp2 from Skp1 and accumulated its substrates p27 [18] and E-cadherin [34] which were involved in regulation of G2-M progression [18] (Figures 3, 4). These results suggest that cyclin B1, p27 and E-cadherin may play a role in 6-OAP-induced prometaphase arrest (Figure 5L), and other NIPA substrates critical to G2-M progression need to be uncovered.

Anti-mitotic drugs have been proven to be one of the most successful chemotherapeutics used for anti-cancer treatment, and microtubule-targeting agents taxanes and vinca alkaloids remain to date the most reliable antimitotics [44]. To bypass the adverse effects including myelosuppression and neurotoxicities, novel targets for the development of antimitotics have been investigated. Evodiamine induced mitotic arrest and down-regulated Skp2 and NIPA (Figure 1K, 1L), but it also induced polymerization of tubulin [45]. 6-OAP did not interfere with microtubule (Figure 4D), therefore its adverse effect might be low, and our *in vivo* experiments confirmed this possibility (Figure 5). 6-OAP significantly suppressed tumor growth in two murine models for lung cancer (Figure 5), and at a dose (600 mg/kg) 30 times higher than the treatment dosage (20 mg/kg) it did not exhibit obvious acute toxicity (Figure 5). 6-OAP also showed anti-myeloma activity with low cytotoxicity and favorable pharmacokinetic features [43]. Hence, Skp1-targeting represents a promising anti-cancer strategy. Because lung cancer is the No. 1 cancer killer worldwide with an only 15% of five-year overall survival rate for all stages combined [46], a clinical study could be conducted to test 6-OAP's efficacy for this deadly disease. In addition, 6-OAP could be a useful tool to study Skp1 function, since no *Skp1* knockout mouse model is available at present [47].

## MATERIALS AND METHODS

### Ethics approval

The study was approved by the local research ethics committees of all participating sites; all lung cancer samples were collected with informed consent. The animal studies were approved by the Institutional Review Board of Institute of Zoology, Chinese Academy of Sciences. All animal studies were conducted according to protocols approved by the Animal Ethics Committee of the Institute of Zoology, Chinese Academy of Sciences.

### Patient samples

A total of 64 previously untreated NSCLCs from the Department of Thoracic Surgery of the Cancer Hospital, Sun Yat-Sen University were included (Table 1). The patients were diagnosed in the last 5 years and the diagnosis of lung cancer was confirmed by at least 2 pathologists. The tissue samples were taken at the time of surgery and quickly frozen in liquid nitrogen. Immunohistochemistry and Western blot assays were performed as described [48], and the immunoreactivity score (IRS) was calculated as IRS (0–12) = CP (0–4) × SI (0–3), where CP is the percentage of Skp1 positive epithelial cells and SI is staining intensity [49].

### Compounds and molecular docking analysis

Two compound libraries containing 21008 compounds were used for virtual screening. These included 1008 natural compounds and 20000 commercially available small compounds. The 3D structure models of most natural compounds were downloaded from the PubChem compound library. For the remaining natural compounds, 3D models were converted from 2D structures drawn in ChemDraw Ultra and followed by “Minimize Energy” and “Molecule Dynamics” procedure in Chem3D Ultra. The Skp1 model from the crystal structure of Skp1-Skp2-Cks1 in complex with a p27 peptide (PDB accession 2AST) was used as the receptor in the molecular docking. Virtual screening by rigid docking was carried out using Autodock Vina [31]. Receptor and ligands were prepared by AutoDock Tools [50] and OpenBabel [51], respectively. The parameters exhaustiveness and num_modes were set as 50 and 100, respectively. The compounds were also analyzed by the Lipinski's rule of five [33] and ADME/Tox prediction using Schrödinger QikProp program [52].

6-OAP was isolated from medicinal plant *Centipeda minima* by our chemistry group, and the purity of this compound reached 99.5% [53]. Labeling of 6-OAP was performed by Boshixing Synthetic Technologies, Inc. (Shenzhen, China): biotin was condensed with 2-aminoethylthiol using dicyclohexylcarbodiimide to afford the corresponding amide, the thiol group of which was added to the enone moiety of 6-OAP to provide the desired biotinated 6-OAP. The identity of the compound was verified using mass spectrum and nuclear magnetic resonance, and the purity of biotin-6-OAP was determined by HPLC.

The 3-(4,5)-dimethylthiahiazo (-z-y1)-3, 5-di-phenytetrazoliumromide (MTT), cycloheximide (CHX), MG-132 and biotin were purchased from Amresco Inc. (Solon, OH) and Sigma-Aldrich (St. Louis, MO), respectively. Bio-6-OAP was synthesized by Boshixing Synthetic Technologies, Inc. (Shenzhen, China).

### Antibodies

The antibodies used in this study were as follows: anti-Cyclin B1, anti-pCdc2 (Tyr15), anti-Cdc2, anti-ubiquitin, anti-E-cadherin, anti-NIPA, anti-Cul1, goat anti-rabbit IgG-HRP and goat anti-mouse IgG HRP antibody (Cell Signaling Technology, Beverly, MA); anti-α-tubulin and anti-Skp1 (Santa Cruz Biotechnology, Santa Cruz, CA); human anti-centromere proteins serum (Antibodies Inc., Davis, CA); anti-Flag M2, and anti-β-Actin (Sigma).

### Cell culture, cell viability and cell cycle

The lung cancer A549, NCI-H1975, NCI-H460 and NCI-H292 cell lines, normal human bronchial epithelial cells (16HBE), embryonic lung fibroblast HLF and embryonic kidney 293T cells were cultured as described [54]. The cells were tested and authenticated by the Goldeneye™20A STR methods. For MTT assay, exponentially growing cells (1 × 10^4^ in 180 μl) were plated in 96-well microplates, and 20 μl of 10 × drug was added to each well. Cells were incubated with or without 6-OAP for 44 h, followed by co-incubation with 100 μg MTT for 4 h. The microplates were centrifuged, supernatants were removed, and MTT formazan crystals were resolubilized by adding 150 μ1 dimethylsulfoxide (DMSO) to each well. Microplates were then agitated on a plate shaker for 5 min, and absorbance was measured using a multiplate reader at the wavelength of 570 nm [55]. For cell cycle analysis, cells were synchronized to G1/S boundary by a double-thymidine block [56], and then exposed to 6-OAP for indicated time points. Cells were harvested, fixed with 70% cold ethanol, incubated with RNase, and stained with propidium iodide [57]. Cell cycle distribution was analyzed by flow cytometry and CellQuest software. Asynchronous cells upon 6-OAP were also analyzed. Clonogenic assay [48], immunofluorescence staining [54] and analysis of cold-stable microtubules [35] were performed as described.

### Plasmids, siRNA and transfection

The coding sequence of wild-type or mutant *Skp1* and *NIPA* was cloned into the pcDNA3.1-flag expression vector (Invitrogen, Carlsbad, CA, USA). The siRNAs targeting candidate genes were designed and synthesized by Shanghai GenePharma Co., the siRNA sequences were as follows: 5′-CGCAAGACCUUCAAUAUCATT-3′ (Skp1 siRNA1), 5′-CCAAUAUGAUCAAGGGGAATT-3′ (Skp1 siRNA2), and 5′-GUCCACGUCACUGCCUGUATT-3′ (NIPA siRNA). Using lipofectamine 2000 (Invitrogen, California, USA), A549 cells were transfected with 100 nM siRNA. And 48 h later, the cells were treated with or without 6-OAP at 7.5 μM for indicated time points. The cells were then harvested for cell cycle analysis, immunofluorescence staining, or lysed for Western blotting.

### Immunoprecipitation and streptavidin agarose affinity assay

Cell pellets were lysed, lysates were centrifuged, the supernatant was incubated with indicated antibodies overnight at 4°C, after which protein A/G Plus beads (Santa Cruz Biotechnology) were added and incubated at 4°C for 4 h. The beads were washed 4 times in NETN buffer (1% NP-40, 2mM EDTA, 40 mM Tris-Hcl, 137mM NaCl, pH 7.4), resuspended in SDS-PAGE loading buffer and boiled for 5 min. For Streptavidin agarose affinity assay, cells upon Bio-6-OAP were lysed, the lysates were incubated with streptavidin agarose, washed and boiled in SDS-PAGE loading buffer. For 6-OAP competition, the cell lysates were pretreated with 6-OAP (100 μM) for 1 h, followed by 50 μM Bio-6-OAP treatment for 3 h at 4°C, and streptavidin agarose affinity assay were performed. Western blot assays were performed as described [54].

### Western blot and immunohistochemistry assays

For Western blot assay, tissue specimens were ground in liquid nitrogen-cooled mortar, tissue powder was suspended in lysis buffer (50 mM Tris-HCl (pH 7.4), 150 mM NaCl, 1% triton X-100, 1% sodium deoxycholate, 0.1% SDS, 1 mM Na3VO4, 1 mM NaF, 1 mM EDTA, 1 mM PMSF, complete protease inhibitor cocktail) and cleared by centrifugation. Equal amounts of samples were separated by SDS-PAGE, transferred on to nitrocellulose and immunoblotted with indicated antibodies.

Immunohistochemistry assay and scoring of immunoreactivity were performed as described [58]. Formalin-fixed, paraffin-embedded lung cancer tissue specimens (5 μm) were deparaffinized and subjected to a heat-induced epitope retrieval step for 2 min. The H_2_O_2_ (3%) was used to block endogenous peroxidase activity for 10 min. The sections were then washed with PBS. The rabbit polyclonal anti-Skp1 antibody was applied to the slides at a dilution of 1:500 at 4°C overnight. Detection was achieved with the Immunohistochemistry SP kit (pv-6001) (Zhongshan Golden Bridge Biotechnology Co., Ltd, Beijing, China) according to the manufacturer's protocol. Sections were colored with 3, 3′-diaminobenzidine (DAB) and counterstained with hematoxylin, dehydrated, treated with xylene, and mounted. Skp1 protein levels were scored as described [49].

### Murine models

The mice were bred and maintained in a specific pathogen-free environment. The nude mice were injected subcutaneously with H1975 cells (2 × 10^6^) into the right flank. When tumor reached a palpable size, animals were randomized into 4 groups (*n* = 10 for each group) and injected with 6-OAP (10, 15, 20 mg/kg/day) or vehicle (5% Cremophor/5% Ethanol in normal saline) intravenously for 24 days. The animals were sacrificed after the last injection and the tumors were excised; cells were separated and lysed for Western blotting. SCID/beige mice were injected with A549-luciferase (A549-Luc) cells (1 × 10^6^) via tail vein, and a week later the mice were randomized into 2 groups to receive treatment with intravenous injection of vehicle (*n* = 6) or 6-OAP at 20 mg/kg (*n* = 8; once every two days for 30 days). The mice were imaged by IVIS Spectrum at day 40, and were euthanized by cervical dislocation when they became moribund.

Kunming mice (5-week old) were randomized into 4 groups (*n* = 10 for each group) and injected with 6-OAP (225, 375, and 600 mg/kg) or vehicle intravenously at the first day. The body weights of the mice were measured every day. One week later, the animals were sacrificed and blood samples were collected. Serum was The serum concentrations of aspartate aminotransferase (AST) and creatinine (Cr) were measured using kits from Nanjing Jiancheng Bioengineering Institute (Nanjing, China).

### Statistical analysis

The data are presented as the mean ± SD unless noted otherwise. Differences between data groups were evaluated for significance using Student's *t*-test of unpaired data or one-way analysis of variance and Bonferroni post-test. The tumor volume was analyzed with two-way ANOVA and independent sample *t* test using the software SPSS 12.0 for Windows (Chicago, IL). The survival curves were plotted according to Kaplan-Meier method and compared by log-rank test. *P* values <.05 were considered statistically significant.

### Supplementary data

Supplemental data include 2 tables and 3 figures and legends.

## SUPPLEMENTARY FIGURES AND TABLES



## Abbreviations

6-OAP: 6-O-angeloylplenolin
AST: aminotransferase
BAE: binding affinity energy
CR: creatinine
Cul1: Cullin 1
IRS: immunoreactivity score
NIPA: nuclear interaction partner of ALK
NSCLCs: non-small cell lung cancers
pH3: mitotic-specific phosphorylation of histone 3
SCF: S phase kinase-associated protein 1-Cul1-F-box protein complex
Skp1: S phase kinase-associated protein 1
