# Supplementary material for: Skp1 in lung cancer: clinical significance and therapeutic efficacy of its small molecule inhibitors
Source: Oncotarget. 2015 Oct 12;6(33):34953–67. doi: 10.18632/oncotarget.5547 (PMC4741501; doi:10.18632/oncotarget.5547)
Supplement: Supplementary file 1 [file oncotarget-06-34953-s001.pdf]

SUPPLEMENTARY FIGURES AND TABLES

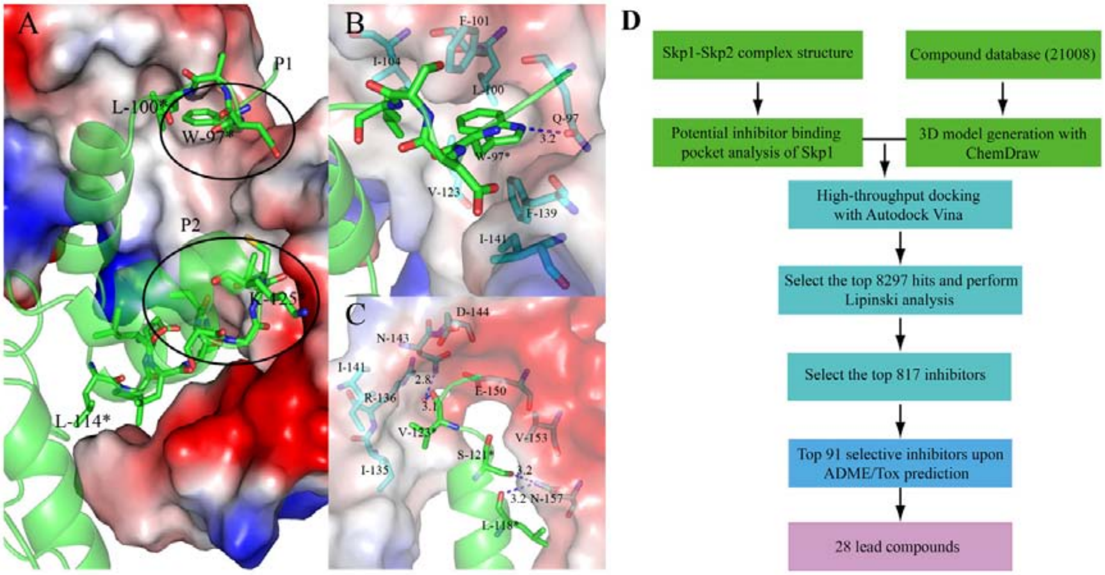

Supplementary Figure S1: Methods for identification of Skp1-targeting agents. A–C. Potential binding pockets (P1 and P2) on Skp1 (PDB code: 2AST). Location of site P1 and P2 on Skp1, the residues of Skp2 inserted into these pockets were shown as sticks. Skp1 and skp2 are shown as surface and cartoon respectively (A) Interactions between Skp2 and the residues at site P1 (B) and P2 (C) of Skp1, respectively. The key residues are shown as sticks and the H-bonds are labeled. The residues from Skp2 are labeled by star. **D.** The high-throughput virtual screening workflow for lead identification.

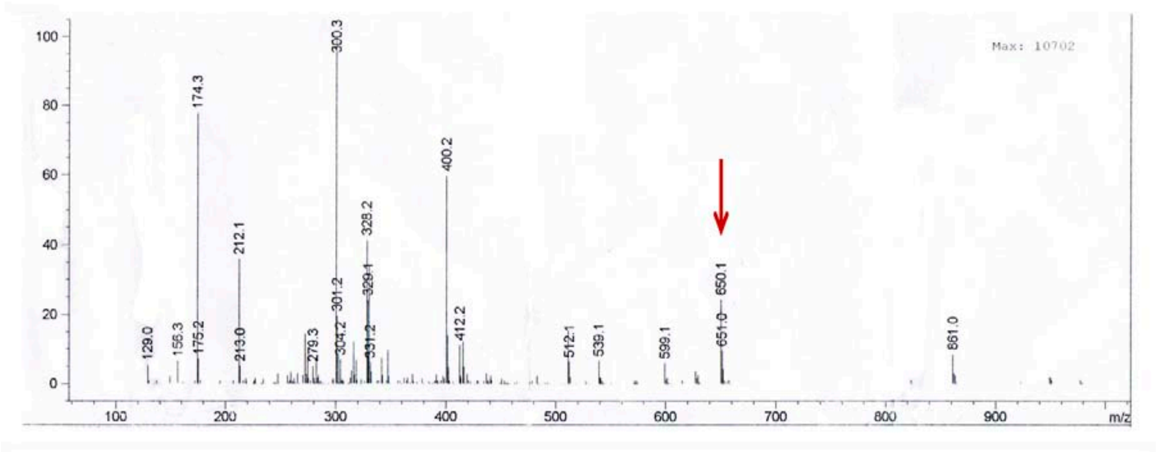

Supplementary Figure S2: Mass spectrum analysis of Biotin-6-OAP.

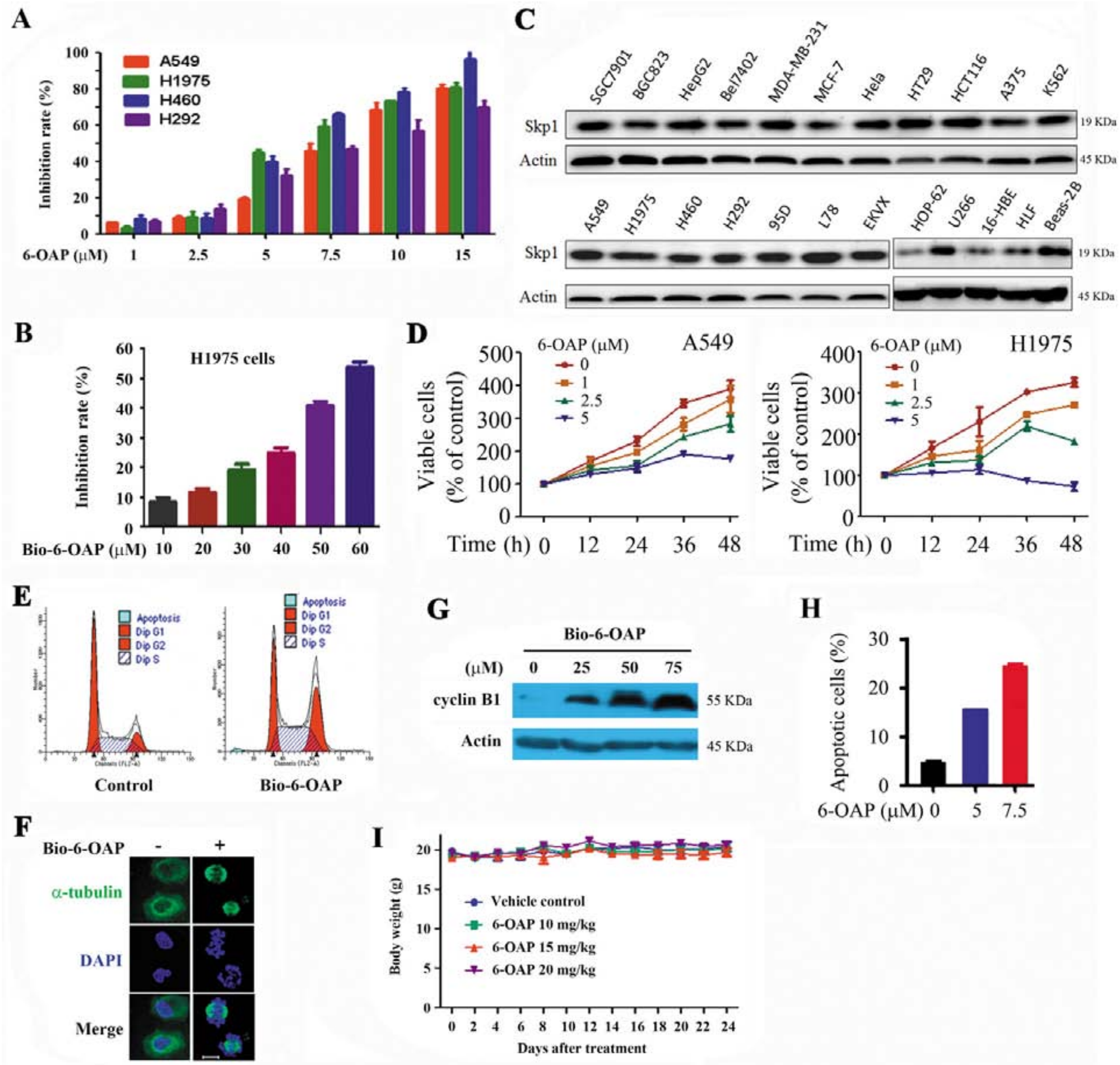

Supplementary Figure S3: Effects of Bio-6-OAP and 6-OAP on lung cancer cells. **A, B.** MTT assays of H1975 cells upon 6-OAP (A) and Bio-6-OAP (B) treatment at indicated concentrations for 48 h. **C.** Western blot analysis of indicated cell lines using indicated antibodies. **D.** The cells were treated with or without 6-OAP, and analyzed by trypan blue exclusion assay. **E.** H1975 cells were treated with 6-OAP for 24 h, fixed with 70% ethanol, and analyzed by propidium iodide staining and flow cytometry. **F, G.** H1975 cells were treated with 7.5 μM Bio-6-OAP for 12 h, and assayed by immunofluorescence labeling with anti-α-tubulin antibody and DAPI (F), or lysed for Western blot assays. **H.** A549 cells were treated with 6-OAP for 24 hours, and assessed by Annexin V/flow cytometry. **I.** Treatment with 6-OAP did not significantly affect body weight of the nude mice.

Supplementary Table S1: Effects of the Skp1-targeting compounds on cancerous and normal lung epithelial cell lines

No.	Compounds	Chemical structure	BAE (kcal/mol)	GI50 (μM)						
				A549	H1975	H460	BGC823	MCF7	BEL7402	16HBE
1	E766–0019	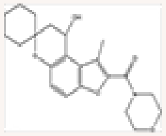	−7.8	>100	>100		>100			
2	8610–0668	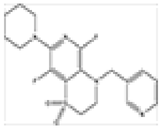	−7.6	>100	>100		>100			
3	Liriodenine	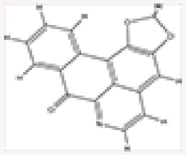	−7.6	3.004	8.115	3.714	2.776	3.853	2.031	8.81
4	E754–0530	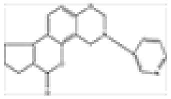	−7.6	>100	>100		>100			
5	F059–1240	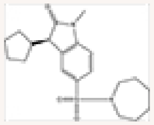	−7.6	11.3	>100		14.3			
6	Evodiamine	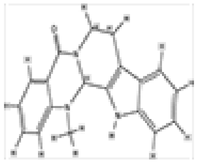	−7.5	1.325	14.219	3.886	0.451	0.506	0.196	> 20
7	J005–0626	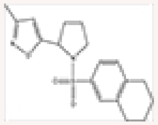	−7.5	>100	96.3		22.4			

(*Continued*)

No.	Compounds	Chemical structure	BAE (kcal/mol)	GI50 (μM)						
				A549	H1975	H460	BGC823	MCF7	BEL7402	16HBE
8	6-OAP	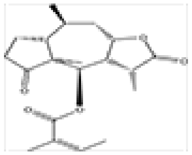	−7.4	2.863	2.76	4.18	3.502	2.919	2.381	8.76
9	C276–0251	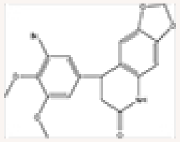	−7.3	6.43	8.92		3.11			4.83
10	C276–0011	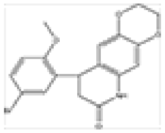	−7.2	1.67	1.62		1.61			4.03
11	α-lapachone	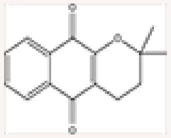	−7.1	6.85	2.22		>100			4.84
12	C276–1418	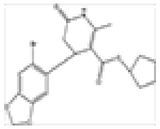	−7.4	>100	>100		>100			
13	D470–0404	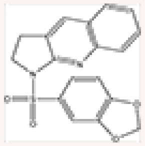	−7.4	>100	>100		43.1			
14	E851–1899	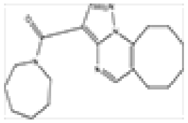	−7.4	>100	>100		26.8			
15	F059–1250	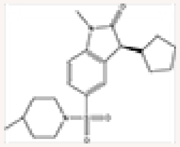	−7.4	28.2	>100		40.9			

(*Continued*)

No.	Compounds	Chemical structure	BAE (kcal/mol)	GI50 (μM)						
				A549	H1975	H460	BGC823	MCF7	BEL7402	16HBE
16	J005–0627	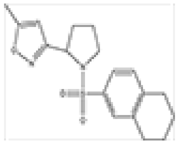	−7.4	>100	89.4		>100			
17	C301–8636	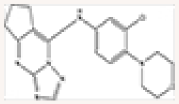	−7.3	>100	39.1		>100			
18	D398–0606	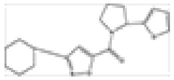	−7.3	>100	>100		>100			
19	G702–3319	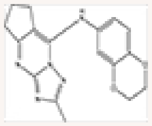	−7.3	>100	>100		>100			
20	G702–0820	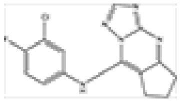	−7.2	>100	>100		>100			
21	K261–0992	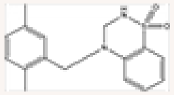	−7.2	>100	>100		>100			
22	K261–0993	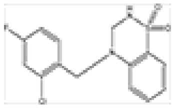	−7.2	>100	>100		>100			
23	2360–0370	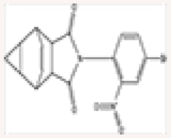	−7.1	15.9	>100		20.5			
24	4607–1348	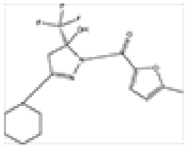	−7.1	>100	>100		>100			

(*Continued*)

No.	Compounds	Chemical structure	BAE (kcal/mol)	GI50 (μM)						
				A549	H1975	H460	BGC823	MCF7	BEL7402	16HBE
25	D150–0115	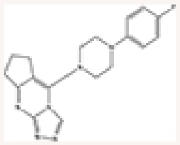	−7.1	>100	>100		>100			
26	D322–0003	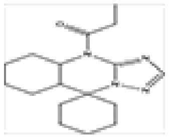	−7.1	>100	>100		>100			
27	G702–0783	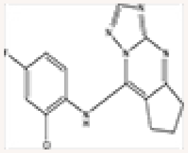	−7.1	>100	>100		>100			
28	K261–1745	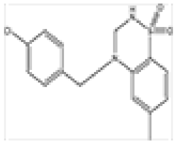	−7.1	>100	>100		>100			

Supplementary Table S2: The relative Skp1 expression and GI50s of 6-OAP on the cell lines

Cell lines	Cell types	Skp1/Actin	GI50s
A549	lung cancer	1.23	2.86
H1975	lung cancer	0.95	2.94
H460	lung cancer	0.91	3.91
H292	lung cancer	0.92	3.21
95D	lung cancer	1.25	3.00
L78	lung cancer	1.53	3.63
EKVX	lung cancer	1.13	2.29
HOP-62	lung cancer	0.72	6.58
U266	myeloma	1.24	2.51
SGC7901	gastric cancer	0.93	5.00
BGC823	gastric cancer	0.90	4.33
HepG2	liver cancer	0.95	9.31
Bel7402	liver cancer	0.93	3.02
MDA-MB-231	breast cancer	0.95	5.87
MCF-7	breast cancer	0.87	3.23
HeLa	cervical cancer	0.96	3.96
HT29	colon cancer	1.52	2.72
HCT116	colon cancer	1.42	2.44
A375	melanoma	0.98	2.80
K562	leukemia	1.05	3.66
UO-31	kidney cancer	0.45	7.68
HLF	normal human embryonic lung fibroblasts	0.88	4.90
16HBE	normal human bronchial epithelial cells	0.81	8.76
